# Metagenome-Assembled Genomes from Photo-Oxidized and Nonoxidized Oil-Degrading Marine Microcosms

**DOI:** 10.1128/mra.00210-23

**Published:** 2023-05-10

**Authors:** Shelby J. Barnes, Raven C. Althouse, Bianca F. Costa, Boyan Hu, Maxim Kovalev, Timur Kulik, Yu-Tung Lee, Meredith C. Moore, Emily Peng, Jing Yao Pook, Akshita Sharma, Celia Wood, Eric A. Webb, Hannah Sterling, Christoph Aeppli, J. Cameron Thrash

**Affiliations:** a Department of Biological Sciences, University of Southern California, Los Angeles, California, USA; b Department of Civil and Environmental Engineering, University of Southern California, Los Angeles, California, USA; c Department of Biology, Marine Biology, and Environmental Science, Roger Williams University, Bristol, Rhode Island, USA; d Bigelow Laboratory for Ocean Sciences, East Boothbay, Maine, USA; Montana State University

## Abstract

We performed deep metagenomic sequencing on hydrocarbon-degrading marine microcosms designed to experimentally determine the effect of photo-oxidation on oil biodegradation dynamics. Assembly, binning, and dereplication yielded 73 unique metagenome-assembled genomes (MAGs) from 6 phyla, of which 61 are predicted to be over 90% complete.

## ANNOUNCEMENT

Microorganisms are the first responders to oil spills and are ready degraders of hydrocarbon compounds (1–3). However, as oil accumulates in surface slicks, photo-oxidation can lead to increased toxicity and affect lability and dispersant effectiveness (4–6). To investigate the interactions between photo-oxidation and the microbial biodegradation of oil, we monitored microcosms over a 4-week period measuring microbial communities and hydrocarbon concentrations. We additionally sampled two treatments for deep metagenomic sequencing to investigate the metabolic potential of organisms enriched in photo-oxidized versus nonoxidized oil treatments.

Triplicate 20-L seawater microcosms were incubated with either oil (“Oil”; Louisiana light sweet crude oil, 25 to 30 ppm) or photo-oxidized oil (“OxHC”; photo-oxidized in a SUN Xe-1 Q-Lab [Westlake, OH] solar simulator as 100-μm films on a temperature-controlled glass plate for 24 h, 30 ppm) or left unamended at room temperature with constant stirring. Oil and OxHC were prepared as a high-energy water accommodated fraction (HEWAF) by blending 500 mg/L in seawater (both oil and seawater were sterile). HEWAF was added at 15% to 5 μm filtered seawater collected from the Bigelow Laboratory for Ocean Sciences pier. Microcosm samples (500 mL) for metagenomic sequencing were taken at the 4-week time point and filtered through 0.2-μm filters (Sterlitech Corp.), and DNA was extracted using a modified phenol-chloroform method (7).

Library preparation and sequencing were performed by Novogene, Inc., using a NEBNext Ultra DNA library kit and an Illumina NovaSeq instrument generating paired-end 150-bp reads. Four samples (two replicates each from Oil and from OxHC treatments) were sequenced generating 41,840,906, 54,525,390, 52,487,390, and 51,607,990 reads for Oil R1, Oil R2, OxHC R1, and OxHC R2, respectively. Adaptors were removed and reads were initially quality filtered using fastp v0.23.1 (8) for reads with N of >10% and Qscore of ≤5 over greater than 50% of the read by Novogene.

MAGs were reconstructed by 11 students of the University of Southern California (USC) BISC 419L course Microbiology for a Sustainable Future. Each sample was assembled and binned separately and independently by either 3 or 4 students using KBase (9) with the “Genome Extraction from Shotgun Metagenome Sequence Data Narrative” (https://narrative.kbase.us/narrative/132113). Reads were evaluated with FastQC v0.11.9 (10), trimmed with Trimmomatic v0.36 (11), and assembled with MetaSPAdes v3.15.3 (12), MEGAHIT v1.2.9 (13), and IDBA-UD v1.1.3 (14). The best assembly was chosen using the Kbase module “Compare Assembled Contig Distributions” v1.1.2 based on maximum contig length and contig *N*_50_ value. Contigs were then binned into MAGs with MaxBin 2.0 v2.2.4 (15), MetaBAT 2 v1.7 (16), and CONCOCT v1.1 (17), and the best MAGs were selected using DASTool v1.1.2 (18). Externally from KBase, all MAGs from each student assembly were run through CheckM2 v1.0.0 “predict” (19), and these statistics were fed into the dRep v3.4.0 “dereplicate” tool (20) to remove highly similar MAGs. Coverage was computed with CoverM v0.4.0 (https://github.com/wwood/CoverM) with bwa-mem and -m mean, and MAG taxonomy was assigned using GTDB-tk v2.1.1 classify_wf (21). Finally, MAGs were annotated with the NCBI Prokaryotic Genome Annotation Pipeline (22). Default settings were used for all software unless otherwise noted. Median estimated MAG completion was 97.78% (min, 77.23%; max, 100%). Assembly and binning information, along with genome statistics and taxonomy for the 73 dereplicated MAGs, are available in Table S1 (see Table S1 at https://doi.org/10.6084/m9.figshare.22696282).

### Data availability.

This metagenomic sequencing project has been deposited in DDBJ/ENA/GenBank under BioProject no. PRJNA938176. Individual MAG BioSample and accession numbers can be found in Table S1 (see Table S1 at https://doi.org/10.6084/m9.figshare.22696282). Raw reads have been deposited at the Sequence Read Archive as follows: SRX19489715 (Oil_R1), SRX19489716 (Oil_R2), SRX19489717 (OxHC_R1), and SRX19489718 (OxHC_R2).
